# Phospholipid homeostasis, membrane tenacity and survival of Mtb in lipid rich conditions is determined by MmpL11 function

**DOI:** 10.1038/s41598-018-26710-z

**Published:** 2018-05-29

**Authors:** Ankur Bothra, Prabhakar Arumugam, Vipul Panchal, Dilip Menon, Sonali Srivastava, Deepthi Shankaran, Ananya Nandy, Neetika Jaisinghani, Archana Singh, Rajesh S. Gokhale, Sheetal Gandotra, Vivek Rao

**Affiliations:** 1 CSIR- Institute of Genomics and Integrative Biology, New Delhi, India; 2 Academy of Scientific and Innovative Research, CSIR-Central Road Research Institute, New Delhi, India; 3 Present Address: National Institute of Immunology, New Delhi, India

## Abstract

The mycobacterial cell wall is a chemically complex array of molecular entities that dictate the pathogenesis of *Mycobacterium tuberculosis*. Biosynthesis and maintenance of this dynamic entity in mycobacterial physiology is still poorly understood. Here we demonstrate a requirement for *M. tuberculosis* MmpL11 in the maintenance of the cell wall architecture and stability in response to surface stress. In the presence of a detergent like Tyloxapol, a *mmpL11* deletion mutant suffered from a severe growth attenuation as a result of altered membrane polarity, permeability and severe architectural damages. This mutant failed to tolerate permissible concentrations of *cis*-fatty acids suggesting its increased sensitivity to surface stress, evident as smaller colonies of the mutant outgrown from lipid rich macrophage cultures. Additionally, loss of MmpL11 led to an altered cellular fatty acid flux in the mutant: reduced incorporation into membrane cardiolipin was associated with an increased flux into the cellular triglyceride pool. This increase in storage lipids like triacyl glycerol (TAG) was associated with the altered metabolic state of higher dormancy-associated gene expression and decreased sensitivity to frontline TB drugs. This study provides a detailed mechanistic insight into the function of *mmpL11* in stress adaptation of mycobacteria.

## Introduction

The mycobacterial cell wall presents as a unique dynamic entity at the interface of Mtb- host interaction and play a determinant role in maintaining cellular integrity in the face of severe stress imparted by the host immune mechanisms^1–3^. Being the primary target for majority of current antimycobacterial compounds, it is logical that mycobacteria have incorporated several efflux pumps in the cell wall^4–8^. Interestingly, Mtb has repositioned members of its cell wall associated RND (Resistance, nodulation and cell division) family of efflux pumps- the MmpS-MmpL systems *viz*. MmpLs- 3,4,5,7,8,10^9–16^ and MmpL11^17,18^ towards the export of complex lipid metabolites for cell wall biogenesis, assembly and sustained maintenance of the mycobacterial cell wall. Except for Mmpl3, the other Mmpls have been shown to be dispensable for *in vitro* growth. Recent studies have attributed an important role for Mtb Mmpl3 as a TMM transporter and also in the intracellular survival of Mtb^19^. On the contrary, MmpL4, MmpL7 and MmpL11 a critical role in the virulence of Mtb in cellular and animal models of infection^20^. Given the putative function in scavenging iron under limiting conditions the essentiality of MmpL4 in *in vivo* growth can be envisaged. MmpL7, involved in the transport of PDIM (phthiocerol dimycocerosates) across the mycobacterial membrane has long been recognised as a virulence determinant in Mtb^21,22^. Its role in establishing a successful role in immune modulation of host macrophage innate response and bacteria mediated host susceptibility to disease is well recognised^23,24^. Recent studies have identified the role of Msm (*Mycobacterium smegmatis*) and Mtb MmpL11 in the transport of a complex cell wall associated mycolic acid containing glycolipids –(mmDAG- monomero-mycolyl DAG and wax esters in Msm and long chain TAGs and wax esters in Mtb) and as an important mediator of Mtb biofilm formation^17,18^. However, the mechanism of this *in vivo* attenuation of the *mmpL11* mutant has not been completely defined. An important role for decreased antigen presentation by ΔMmpl11 mutant to CD8 T cells has been attributed to the lower capacity of the mutant to survive within host cells and granulomas^18^. However, the precise mechanisms that impair the growth of the mutant in host induced stress has not been defined in detail.

Here, we describe the important role of the Mtb MmpL11 in cell wall assembly, integrity and resistance to several external insults. An *mmpL11* deficient Mtb strain (ΔM11) showed decreased survival in the presence of detergents or *cis*-fatty acids. A significant defect to maintain normal fatty acid flux to cardiolipin biosynthetic pathway and its consequent rerouting for triacylglycerol (TAG) biosynthesis in the presence of detergents manifested as a compromised cell membrane of the mutant. The mutant also displayed an altered metabolic state dependent transcriptional signature similar to dormant bacteria thereby highlighting the importance of MmpL11 in the physiology of Mtb. Our results provide a snapshot of the complex molecular machinery of mycobacterial cell wall assembly and maintenance during surface stress and a mechanistic insight into the essentiality of this gene for *ex-vivo* survival of Mtb.

## Results

### Mtb MmpL11 is critical for intracellular and stress dependent growth of Mtb

**Figure 1:**
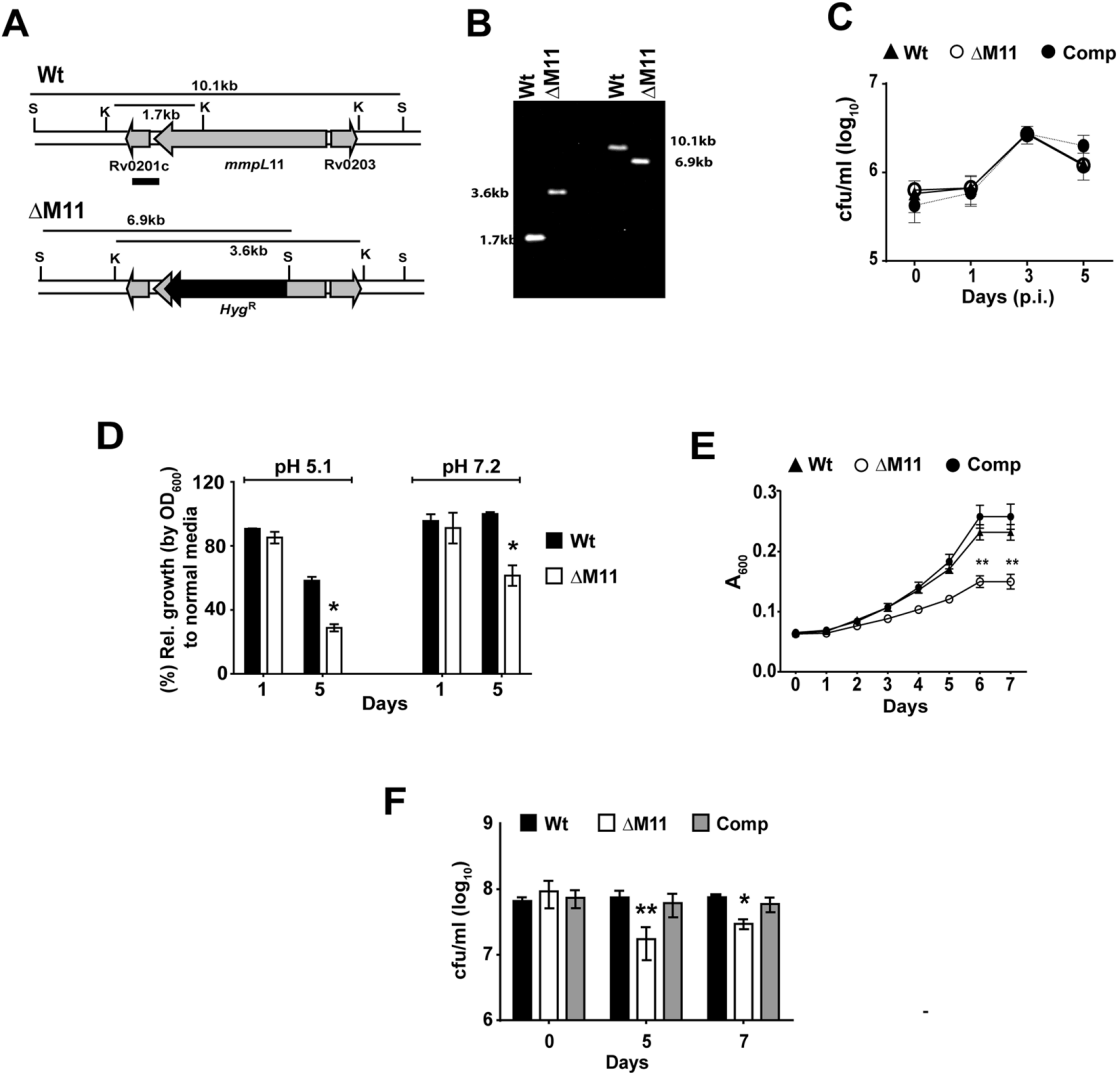
Mtb MmpL11 is essential for the intracellular growth fitness of Mtb: (**A,B**) Confirmation of deletion of *mmpL11* by Southern hybridisation (**B**) according to the strategy described in (**A**). The black bar denotes the probe binding site in the genomic DNA, *kpnI* (k) and *sacI* (s) sites are indicated. (**C**) *Ex vivo* growth of Wt, ΔM11 and Comp in PMA differentiated THP1 macrophages. (**D**) Growth of Wt and ΔM11 in 7H9 media of pH 5.1 and 7.2. (**E**) Growth of Wt and ΔM11 in 7H9 media containing 0.05% Tyloxapol relative to growth in Tween 80 containing media. Values are mean O.D. (A600) ± SD for N = 3 replicates. F) The bacterial numbers at 0, 5, and 7 days of growth in media containing 0.05% Tyloxapol is shown. Values represent the average values ± SD of one of 2–3 replicate experiments.

In an attempt to decipher the function of MmpL11 in Mtb physiology, we constructed an *mmpL11* null mutant (ΔM11) and confirmed the gene deletion by Southern hybridization (Fig. 1A,B) and qPCR; ~40% expression of this gene could be restored in the complemented strain (Comp/ΔM11 + M11) (Fig. S1A).

A previous study had implicated an important role for Mmpl11 in the *in vivo* growth of Mtb. To investigate if this essentiality also reflected as a dependence of the gene for pathogen stress survival, we tested *ex vivo* and *in vitro* stress survival of Mtb. In activated THP1 macrophages, loss of *mmpL11* did not affect the growth of the ΔM11 mutant (Fig. 1C). Under most conditions of *in vitro* stress that simulated environments encountered by intracellular bacteria inside host cells, growth of the mutant was not affected viz. different carbon nutritional media or in the presence of reactive oxygen intermediates and reactive nitrogen intermediates (Fig. S1B,C). In contrast, the response to acidified media varied between the Wt and ΔM11 strains. When grown in media supplemented with 0.05% Tyloxapol at pH 5.1 for 5 days, ΔM11 growth was decreased by 1.6–2 folds in comparison to the Wt (Fig. 1D). Interestingly, this growth defect of ΔM11 was primarily a consequence of 0.05% Tyloxapol in the media as a similar growth defect was also observed at pH 7. Tyloxapol could inhibit growth of the mutant in a dose dependent manner. Both the strains showed comparable growth in 0.02% Tyloxapol (Fig. S1D); at higher concentrations of 0.05% and 0.1% Tyloxapol, however, the ΔM11 was much slower and could reach only 0.15 and 0.1, respectively (Figs 1E, S1D). In comparison, the Wt showed exponential growth reaching an OD of ~0.25 over 7 days of culture. The growth defect of the mutant was lost in i) the complemented strain and ii) in media supplemented with ADC (Fig. S1E). This decreased growth of the mutant was a consequence of a 2.4–4.5 folds decrease in bacterial numbers as compared to the Wt in 0.05% Tyloxapol (Fig. 1F). Interestingly, ΔM11 was inhibited by other detergents like Triton X-100 and IGEPAL CA-630 (NP-40) in the growth media, suggesting an important function of Mtb MmpL11 in response to detergents (Fig. S1F).

### Mtb MmpL11 is critical for maintenance of cell wall architecture and function in detergent stress

**Figure 2:**
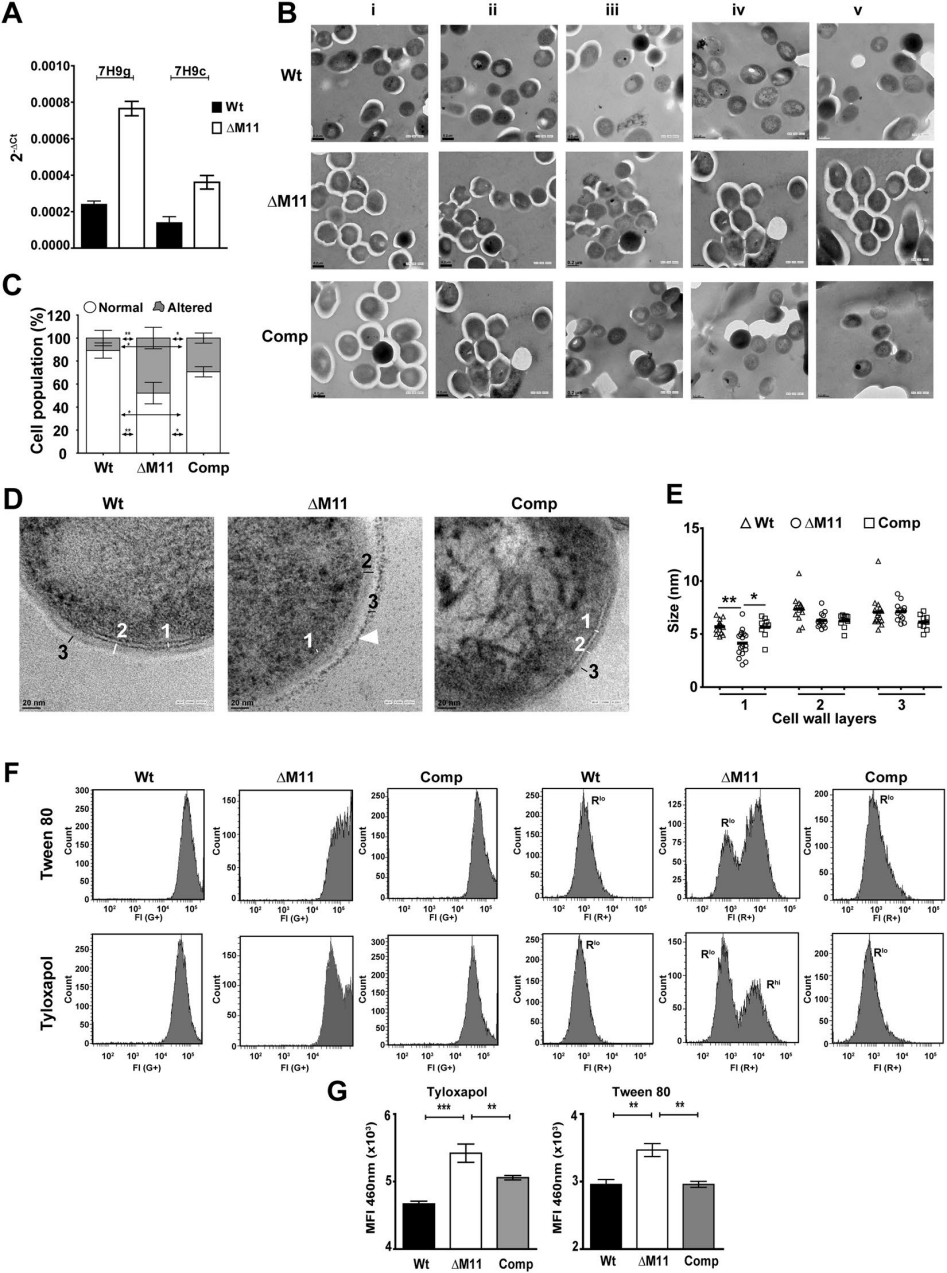
Loss of *mmpL11* in Mtb results in changes of the gross cell wall ultrastructure and in membrane integrity in Tyloxapol: (**A**) Expression of *sigE* gene in the Wt and mutant strain by qRT PCR in media containing 0.05% Tyloxapol - 7H9g (7H9 containing 0.5% glycerol) and 7H9c (0.5% glycerol with ADC). The fold change in Ct values with respect to 16 s rRNA is depicted. (**B**,**D**) Representative TEM micrographs (5 different fields are shown as i-v) of Wt, ΔM11 and Comp strains grown in media containing 0.05% Tyloxapol. Scale bars of 0.2 µm (**B**) and 20 nm (**D**) are shown. (**C**) Quantitation of cells in the samples with intact morphology (round) and rhomboid morphology (outlines) from 3 independent experiments. (**E**) Thickness of cell wall layers of Wt, ΔM11 and Comp Mtb strains. Each symbol represents the micrograph of one cell, median values are depicted. (**F**) Analysis of membrane polarity in media containing either 0.05% Tween 80 or Tyloxapol by DiOC_2_(3) staining and FACS. Fluorescence intensities (FI) at 497 nm (G+) and 615 nm (R+) are represented as histograms. R^lo^ and R^hi^ represent the tow populations with low and high red intensities, respectively. (**G**) Uptake of Hoechst-33342 by the Mtb strains. Data represents mean fluorescence intensities (MFI) ± SD values of triplicate wells in a representative experiment of n = 3.

Detergents are capable of modifying the cell wall composition primarily resulting in surface stress to the bacteria^25–27^. The alternate sigma factor, SigE, is recognised as one of the primary response regulator of surface stress in Mtb^28^. In order to understand the basis of Tyloxapol induced growth defect in the mutant particularly, we analysed *sigE* expression in the different strains. In Tween 80 containing media, we could not detect any expression in either the Wt or mutant strains. Expression however, was significantly augmented (4 folds higher than Wt) in the mutant in response to the presence of Tyloxapol in the growth medium suggestive of surface stress to the bacterium. This increase was offset by the presence of media containing ADC as the supplement (Fig. 2A).

This prompted an evaluation of the overall cell structure of Mtb following Tyloxapol treatment by Transmission electron microscopy (TEM). In media containing Tween 80, majority of Wt and ΔM11 cells (>95%) displayed normal morphology (Fig. S1A–C). However, substitution with Tyloxapol induced significant changes in cellular morphology in Mtb (Fig. 2B). Nearly 2–4-fold greater numbers of ΔM11 had lost their rounded normal morphology and were seen as irregularly shaped cells in comparison to the Wt and Comp (49%, 11% and 29%, respectively) in Tyloxapol (Fig. 2C). The effect of Tyloxapol on the ultrastructure of mutant cell wall was more evident at a higher magnification wherein the innermost phospholipid bilayer (layer1) was the most affected (Fig. 2D,E). In both the Wt and complemented strains treated with Tyloxapol, the plasma membrane thickness ranged from 6.2–9.2 nm, in contrast, this layer was significantly thinner (between 3.3 to 6.6 nm) for the ΔM11 (Fig. 2D,E). In addition, an extra electron dense layer was observed only in the periphery of the mutant strain (white arrowheads, Fig. 2D).

Since membrane architecture dictates membrane polarity^29^, we tested if Tyloxapol induced surface alterations affected the polarity of Mtb using DiOC_2_(3). As seen in Fig. 2F, both Wt and Comp showed a single uniformly green fluorescent population (em497nm, G^+^) in Tween 80 that was unaltered by Tyloxapol treatment. However, mutant cells displayed characteristics of significant membrane hyperpolarization with two distinct populations (em615nm, R^+^) - i) strongly fluorescent R^hi^ and ii) R^lo^ that was also seen in the Wt and Comp strains in the presence of Tween 80 and Tyloxapol. Additionally, G + population in the mutant displayed a more diverse population indicative of variable dye accumulation in the cells. With a direct correlation between alterations of zeta potential and changes in membrane permeability^30^, we tested the effect of Tyloxapol on mutant membrane permeability by using the cell permeable DNA binding Hoechst-33342 dye. As observed in Fig. 2G, nearly 2-fold increase in the intracellular accumulation of the dye was seen in the Tyloxapol treated ΔM11 cells as compared to the Wt; the increased accumulation returned to the normal levels in the complemented strain. Moreover, loss of Mmpl11 in the mutant resulted in an altered architecture and permeability with hyperpolarisation of the plasma membrane even under normal growth conditions. Taken together, these studies indicated that Mmpl11 was essential for maintaining membrane architecture and functionality in both normal and stress dependent physiological conditions.

### Loss of MmpL11 severely affects the cardiolipin content of Mtb in the presence of Tyloxapol

**Figure 3:**
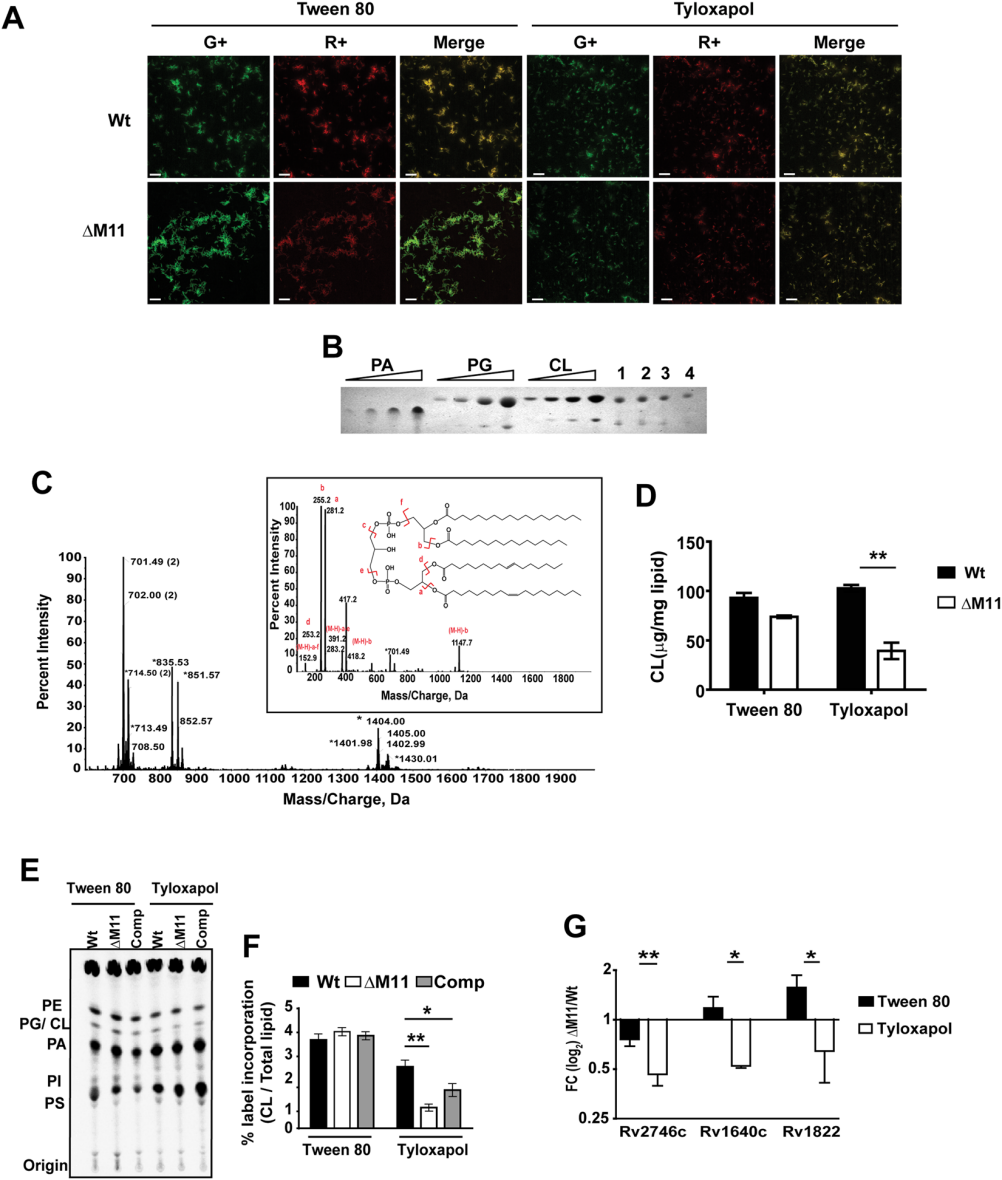
Requirement of MmpL11 for maintaining the Mtb phospholipid composition under stress. (**A**) Analysis of phospholipid composition of Wt and ΔM11 strains grown in the presence of either 0.1% Tween 80 or Tyloxapol for 24 hr by fluorescence microscopy at em529 nm (G+) and em605 nm (R+) and a merge of the two channels are shown. (**B,C,D**) -Quantitation of CL levels by densitometry-B) Separation of CL in fractionated lipids of Mtb by TLC (CHCl_3_:CH_3_OH:H_2_0::65:25:4), samples 1 and 3 represent Wt, 2 & 4- ΔM11 in media containing Tween 80 (1, 2) or Tyloxapol (3, 4), Purified PG, PE, CL were used at increasing concentrations as standards for internal reference; (**C**) Confirmation of purified CL fraction by Tandem ESI- MS analysis. The MS1 profile of m/z 1404.00 corresponding to CL with the MS2 fragmentation profile and a putative structure shown in inset; (**D**) Estimation of CL concentration by densitometry is represented as mean ± SD for a representative of 3 biological replicate experiments. (**E**,**F**) Analysis of ^14^C-oleate incorporation into Mtb lipids- Separation of phospholipids by TLC (**E**) and quantitation by liquid scintillation count is represented as average relative counts of CL ± SD for a representative of 3 biological replicate experiments. (**F**,**G**) Analysis of expression of genes involved in CL biosynthesis of Mtb by qPCR. Ratio of expression in the mutant with respect to Wt is depicted as relative transcripts ΔM11/Wt (fold change in mutant relative to Wt).

In order to investigate the molecular basis for changes in ΔM11 membrane permeability, we analysed the phospholipid profile of Mtb strains by nonyl acridine orange (NAO) staining (Fig. 3A). Presence of Tyloxapol instead of Tween 80 in the media resulted in a significant increase in the red fluorescence **(**R^+^) in the Wt strain by ~30%. In contrast, there was a reduction in R^+^ of ΔM11 cells (~20%) in Tyloxapol. Overall, this significant decrease was suggestive of a reduced cardiolipin (CL) content of the mutant (Fig. S1A). To estimate the decrease in CL levels of the mutant, total lipids were subjected to SPE (Aminopropyl-bonded silica gel cartridge) fractionation and quantitated by TLC (Fig. 3B) and confirmed the identity of CL in this lipid fraction by ESI-MS (Fig. 3C). As expected, the major ion of m/z 1404.00 correlated with the CL molecule composed of C18:0, C18:1, C16:0, C16:1 by MS2 (inset-Fig. 3C). Densitometric estimation of the CL specific fraction, indicated a ~3 fold decrease in the Tyloxapol treated mutant as compared to the Wt; the levels of CL were similar in the two strains in the presence of Tween 80 (Fig. 3D). Levels of other major phospholipids (PE, PS or PI) were unchanged in the Wt or the mutant in Tween 80 or in the Wt exposed to Tyloxapol.

To establish that the decrease in CL was due to lower *de novo* synthesis in the mutant, we monitored the incorporation rate of ^14^C-oleic acid into the phospholipids pool of the Wt, ΔM11 and complemented Mtb strains (Fig. 3E). Nearly 3.8–4% of the label was seen in the CL fraction of all three strains grown in Tween 80 (Fig. 3F). In contrast, while a marginal decrease in CL was observed for Wt and Comp, ΔM11 showed nearly 4.7-fold lower incorporation of the label in CL (~30% of Wt) specifically on Tyloxapol treatment.

Transcriptional rewiring of metabolite biosynthesis is a well-documented mechanism of stress adaptation in bacteria^31,32^. In an attempt to decipher the basis of lower CL in Tyloxapol treated ΔM11, we analysed the expression of genes involved in plasma membrane biogenesis. As expected, we observed 2.4 folds lower levels of *pgsA2* (Rv1822) expression, the putative CL synthase in ΔM11 in the presence of Tyloxapol (Fig. 3G). In addition, levels of *lysX*- Rv1640c and the putative PGP synthase *pgsA3*- Rv2746c were halved in the mutant, suggestive of a complete down regulation of the PG (Phosphatidyl glycerol)/CL biosynthetic pathway specifically following Tyloxapol treatment of ΔM11 (Fig. 3G).

### CL decrease in mutant is associated with increased accumulation of TAG and dormancy like phenotype

**Figure 4:**
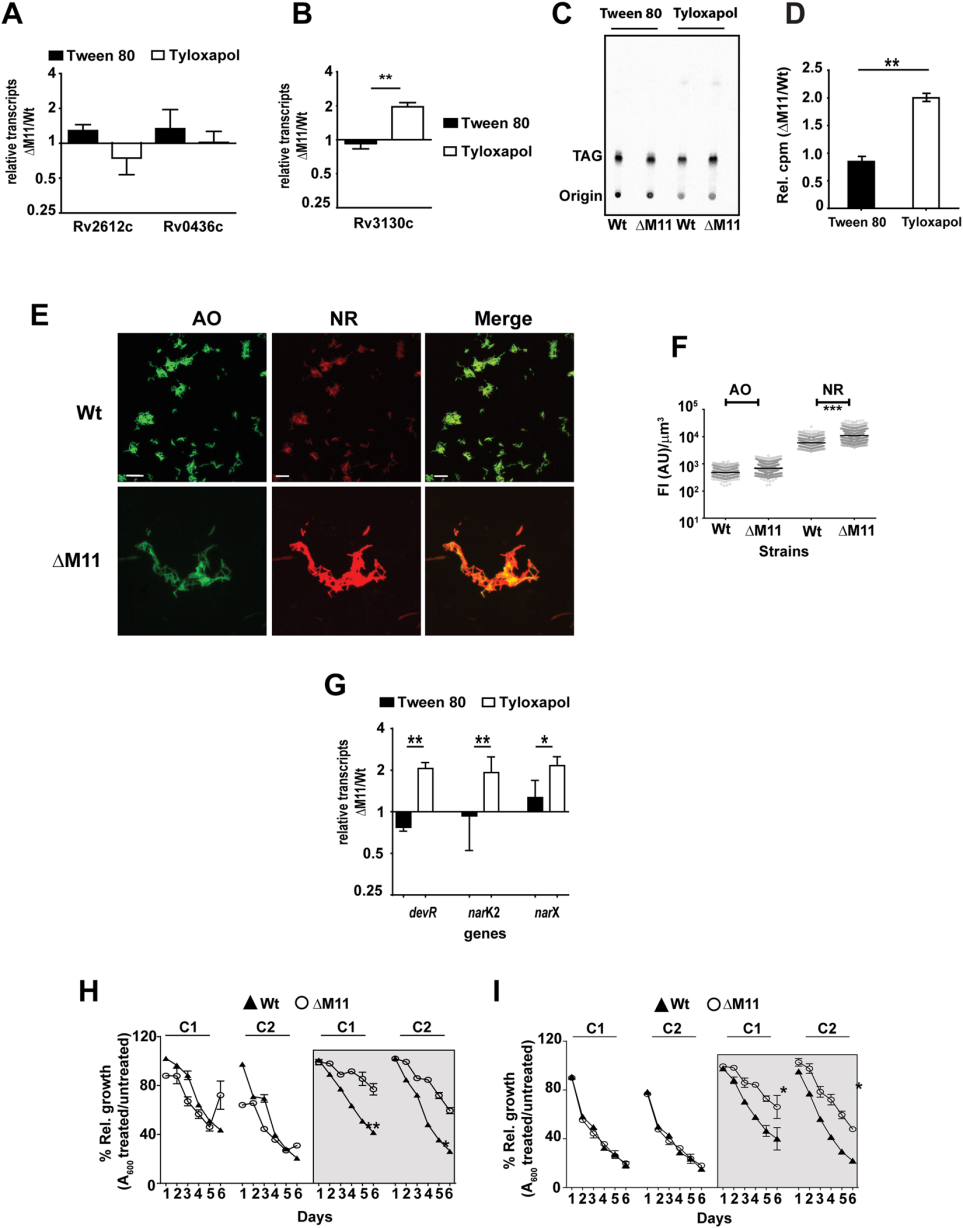
Loss of MmpL11 in Mtb leads to Tyloxapol induced neutral lipid abundance in the cell: (**A,B**) Analysis of expression of Mtb genes by qPCR is represented as relative transcripts ΔM11/Wt (fold change in the mutant with respect to Wt): (**A**) Rv2612c, Rv0436c; (**B**) putative triglyceride synthase *tgs1-* Rv3130c. (**C**,**D**) TLC and radiometric estimation of TAG in Mtb strains in ^14^C-oleate fed logarithmic cultures. (**E**) Representative images for AO and NR staining of Wt and ΔM11 cells grown in the presence of 0.05% Tyloxapol. (**F**) Quantitation of fluorescence intensities (FI) normalized to volume is graphically represented; median values are indicated by the black line for the different groups. (**G**) Analysis of expression of genes involved in the dormancy regulon by qPCR. The ratio of expression in the mutant with respect to Wt is depicted as relative transcripts ΔM11/Wt (fold change in the mutant with respect to Wt). (**H,I**) Growth of Mtb strains in either Tween 80 or Tyloxapol (boxed half) and in the presence of antibiotics as O.D. (A_600_) over 6 days of culture. Two concentrations (62.5 ng/ml-C1 and 125 ng/ml-C2) of rifampicin (H) or streptomycin (250 ng/ml-C1 and 500 ng/ml-C2) (**I**) were used in the study. Values are represented as ratio of mean O.D. (A_600_) in antibiotic relative to no treatment ± SD for one experiment of n = 2.

The gross alteration of the PG/CL biosynthesis in ΔM11 argued well for an alternative rerouting of precursor PA to either the phosphatidyl serine (PS)/phosphatidyl ethanolamine (PE), Phosphatidyl inositol (PI) and TAG biosynthetic pathways^33^. Similar levels of radiolabelled oleic acid incorporation into PE, PS or PI in the three strains (Fig. 3E) substantiated the comparable expression levels of Rv0436c and Rv2612c- putative PS and PI synthases, respectively (Fig. 4A). ΔM11 cells displayed 2.2 fold increase in *tgs1* (Rv3130c) expression, one of the most active triglyceride synthase of Mtb (Fig. 4B) that manifested as a ~2 fold increase in incorporation of ^14^C-oleic acid into the TAG fractions of Tyloxapol treated ΔM11 (Fig. 4C,D). This increment of neutral lipid levels was further supported by the higher Nile red (NR) staining of ΔM11 (Fig. 4E). Incubation with Tyloxapol instead of Tween 80 increased Nile red intensities in both the Wt and ΔM11, however, the increase was much higher for the mutant (2.1×) than the Wt strain (1.3×) (Fig. 4F).

One of the consequences of increased TAG accumulation in Mtb is the acquisition of a dormant metabolic state of mycobacteria^34–36^. Consequent to the increased accumulation of TAG, expression of genes of the Mtb dormancy regulon, *devR*, *narK2* and *narX*, increased by more than 2 fold in the Tyloxapol treated ΔM11 than the Wt strain indicative of an altered metabolic state of the mutant (Fig. 4G). Current anti tubercular drugs like rifampicin and streptomycin target actively growing Mtb and are less effective against dormant bacteria^37^. While both rifampicin and streptomycin could effectively control the growth of Mtb strains in Tween 80 containing media, substitution with Tyloxapol in the media markedly altered the sensitivity of the latter two drugs on the ΔM11 (Fig. 4H,I). Over 6 days of growth, Mtb cultures showed a decline in growth (A_600_) in the presence of the antibiotics in either concentration. Clearly, presence of Tween 80 in the growth media did not differentiate growth profiles of either the Wt or mutant strains. In Tyloxapol while both streptomycin and Rifampicin inhibited the Wt by ~60–80% the effect on ΔM11 was significantly altered (20–45% for the 2 doses of streptomycin and 30–50% for Rifampicin).

### MmpL11 is essential for survival of Mtb in the presence in fatty acid rich conditions

**Figure 5:**
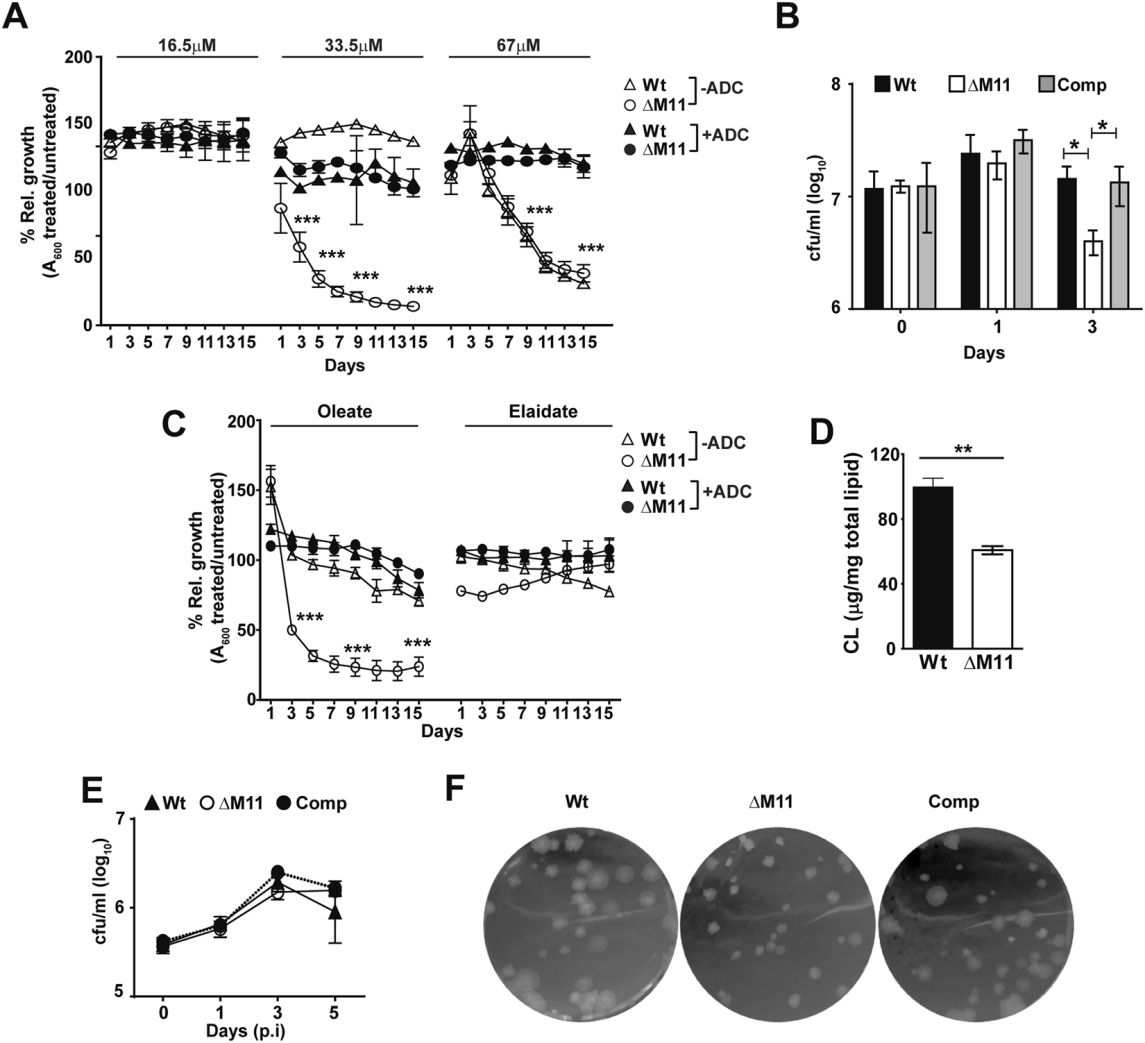
MmpL11 is essential for Mtb to grow in fatty acid rich conditions: (**A**) Bacterial growth in the presence of different concentrations of methyl palmitoleate with (solid symbols) and without ADC (open symbols) for 15 days is depicted as % relative growth mean ± SD of triplicate wells of one of three experiments. Values are represented as ratio of mean O.D. at 600 nm in methyl ester of fatty acids relative to no treatment ± SD. (**B**) The bacterial numbers at 0, 1, and 3 days of growth in media containing 33.5 µM methyl-palmitoleate for the Mtb strains is shown as mean cfu/ml ± SD. (**C**) Growth in the presence of methyl oleate or methyl elaidate with (solid symbols) and without ADC (open symbols) is depicted as the average % relative growth mean ± SD of triplicate wells. (**D**) Quantitation of CL in the lipids of Mtb grown in methyl-oleate for 24 hr by densitometry. The relative amount of CL is represented as mean ± SD for a representative of 2 biological replicate experiments. (**E**) Intracellular growth of Wt, ΔM11 and Comp grown in oleic acid pre-treated differentiated THP1 cells is shown as cfu/ml ± SD of triplicate wells of one of the two experiments. (**F**) A representative image of the bacterial colonies from oleic acid treated THP1 cells at day 1 *p.i*. for the 3 Mtb strains.

A previous study has demonstrated that Mtb grown in the presence of fatty acids shifts to a dormant metabolic state with accumulation of TAGs^38^ as seen in the Tyloxapol treatment of ΔM11. Our observation of Tyloxapol induced dysregulation of CL /TAG biosynthetic machinery and consequent shift in the metabolic state of ΔM11 prompted the evaluation of the effects of fatty acids on this mutant strain. As seen in Fig. 5A, fatty acids were able to restrict the growth of Mtb in a dose and time dependent manner. Both the Wt and mutant were able to withstand methyl-palmitoleate up to a concentration of 16.5 µM. At a higher concentration of 33.5 µM, the ΔM11 failed to grow even after 10 days of culture while the Wt did not show any growth defect at all (Fig. 5A open symbols). Interestingly, as observed with Tyloxapol, this increased susceptibility of the mutant was not observed in ADC containing media (solid symbols). At 67 µM, methyl-palmitoleate prevented the growth of both the Wt and mutant strains equally. We observed a ~4 fold decrease in mutant bacterial numbers suggestive of enhanced susceptibility of ΔM11 to prolonged treatment of fatty acid esters; unfortunately, we could not get single countable colonies at later time points (Fig. 5B). Restoration of *mmpL11* expression in the complement completely abrogated this growth deficiency (Fig. 5A,B).

A similar profile of significantly increased sensitivity of the mutant and rescue by ADC supplementation was observed for oleic acid methyl esters (Fig. 5C). Surprisingly, this growth inhibition property was found only for *cis*- unsaturated fatty acid as the *trans*-unsaturated elaidic acid methyl ester supported similar growth of the Wt and ΔM11 at all concentrations. Moreover, treatment of ΔM11 with methyl-oleate resulted in a significant decrease in CL levels similar to Tyloxapol treated cells establishing that the two molecules imparted similar stress to Mtb (Fig. 5D).

Mtb faces several immune response induced stress inside host cells^39,40^. The gross defect of ΔM11 to grow in fatty acid implicated a possible role of MmpL11 in Mtb survival in a lipid rich cellular milieu like oleic acid fed THP1 cells^41^. Surprisingly, while we did not observe any difference in the Wt or ΔM11 bacterial numbers at any time post infection; growth in this lipid rich environment severely affected the outgrowth ability of the mutant (Fig. 5E). Colonies of ΔM11 were markedly smaller as compared to the Wt after 1 day of infection; the smaller size was completely reversed in the complemented strain (Fig. 5F).

## Discussion

Mtb owes its success as a human pathogen to the manifold strategies employed by the bacterium to counter the host response. The cell wall is a crucial component of the pathogen’s ability to survive intracellularly; it is obvious that Mtb has developed a well-orchestrated array of biosynthetic pathways to maintain the integrity and composition of the cell wall^42,43^. While several reports have aided in delineating the synthetic machinery for the individual cell wall associated components in Mtb over the years^44^, the regulated export of complex lipids and biomolecules to the cell wall from the cellular landscape has not been yet well studied. Recent studies have identified complex lipid transporters involved in maintenance of cell wall architecture in Mtb; several polyketide/ lipids of the cell wall are known cargo for the RND family of proteins – MmpLs in Mtb^16,17^. We decided to evaluate the function of one of the members of this protein family-MmpL11 by genetic disruption in Mtb.

The Mtb ΔM11 was severely compromised in its ability to grow in the presence of detergents like Tyloxapol, Triton X-100 and IGEPAL CA-630. The detergent like activity of lung surfactants in reducing surface tension is well established^45^. Since we did not observe any growth attenuation of the ΔM11 in lung surfactants, physiologically relevant surfactant (Fig. S1B), we assumed that the growth inhibitory effect of detergents was primarily a result of surface stress, a fact that was substantiated by the altered membrane permeability and evidence of severe morphological disturbance of the mutant cell membrane in the presence of Tyloxapol. An inability of the mutant to grow in permissible concentrations of specifically *cis*-unsaturated fatty acids further corroborates the essentiality of MmpL11 in mitigating cell surface stress. Mtb is unable to utilize *cis*-fatty acids directly as a carbon source and depends on it isomerization to *trans*-fatty acids by *echA* gene family^46^. Our observation, that the ΔM11 was capable of growing at lower concentrations and susceptible at higher growth permissible concentrations of fatty acids points towards a defect in its ability to alleviate the toxic effects rather than fatty acid utilization. Interestingly, we observed that the presence of ADC in the medium was sufficient to neutralize the toxic effects of the fatty acid esters. These observations are in line with our data of a dose and time dependent oleic acid sequestering ability of albumin in 7H9 media (Fig. S1C) and a similar study earlier^47^.

With cell surface lipids crucial for growth, stress adaptation and an important role for fatty acids in membrane dynamics of bacteria^27^, we focussed on analysing changes in lipid content of ΔM11 in the presence of Tyloxapol in order to elucidate the molecular basis of its growth deficiency. While we did not observe any change in the major cell wall associated lipids in the mutant strain grown in either Tween 80 or Tyloxapol (Fig. S1D), the phospholipid content was grossly altered. Loss of *mmpL11* in Mtb resulted in decreased levels of cardiolipin in the cell membrane associated with an increase in the cellular TAG levels in both Tyloxapol and fatty acids. Interestingly, one of the modes of unsaturated fatty acid level homeostasis in cells is by incorporation into phospholipids like cardiolipin. Alternatively, bacteria can effectively reroute these toxic fatty acids into the TAG synthesis^38^. A recent study has demonstrated a crucial link between cardiolipin levels and neutral lipid metabolism in *Saccharomyces cerevisiae*^48^. The resultant increase in total TAG levels following a decrease of cellular CL levels in this study only supports our observation with ΔM11 of a similar link between membrane CL levels and intracellular TAG accumulation. Given this scenario it is logical to assume that decreased incorporation of free fatty acids into the cardiolipin pool in the mutant and the associated increase in accumulation of neutral lipids would serve as a salvage pathway for the mutant in Tyloxapol. However, in this situation of an already dysregulated phospholipid metabolism of the mutant, addition of exogenous *cis*-unsaturated fatty acid methyl esters (these can easily enter the bacterial cells) would lead to a further increase in cellular fatty acid levels and thus enhanced sensitivity of the ΔM11. The Wt strain, however, can effectively incorporate the cellular fatty acids into the cardiolipin and TAGs biosynthetic pathway, thus withstanding higher concentrations of exogenous fatty acids in the media.

While it is not clear if entering into a dormant physiological state is the trigger for lipid accumulation or vice versa, there is enough evidence to suggest that dormant Mtb has increased intracellular neutral lipid inclusions^49–51^. The ΔM11 with increased intracellular neutral lipids also displayed higher expression levels of several components of the *devR* regulon correlating with the transcriptional signature of an on-going dormancy like metabolic state^52^. A greater resistance to TB drugs that target actively replicating bacteria like rifampicin and streptomycin further reflected on this altered metabolic state of the ΔM11 mutant. In total, increased expression of tgs1 leading to accumulation of TAG, higher expression of the devR regulon genes in the presence of a lipid rich environment has been associated with increased tolerance of Mtb to INH and Rif ^34^ provides evidence for the phenotype of the ΔM11 mutant in Tyloxapol.

A very recent study by Wright *et al*.^18^ has defined the critical requirement of Mmpl11 in establishing a biofilm growth in Mtb^18^. By using a M11 gene deletion mutant strain they demonstrate that growth is not affected in normal growth conditions; this phenotype is supportive of our findings of no changes in growth of the mutant in normal 7H9 media. In our study, the mutant displayed increased vulnerability in intra-macrophage growth- in THP1 cells, manifesting as smaller outgrown colonies of the mutant as compared to Wt and complemented strains. While the previous study also demonstrates that the mutant does not suffer from growth defects in macrophages in terms of bacterial numbers (CFU) similar to our findings, their study does not reflect on the size of the outgrown colonies as observed in the present study. It is becoming increasingly clear that Mtb infection of macrophages leads to surface stress due to oxidation of cell membrane proteins and lipids of bacteria^53^. Mycobacteria have developed an unique ability for utilization of host lipids by incorporation of the precursors into the bacterial lipids^34^. Mtb driven lipolysis of host lipids by mycobacterial lipases and cutinases is thought to play an important role in this phenomenon^54^. We observed similar levels of radiolabelled fatty acid incorporation by Wt and ΔM11 individually in THP1 macrophages (Fig. S4). One can thus envisage that in macrophages infected with Wt Mtb, the bacteria are effectively able to use host lipids and esterify the consequent fatty acids into either the phospholipid or TAG pool. However, the mutant displayed an altered metabolic state of increased intracellular TAG that might limit its acquisition of host lipids and thus survive in the individual infection model.

**Figure 6:**
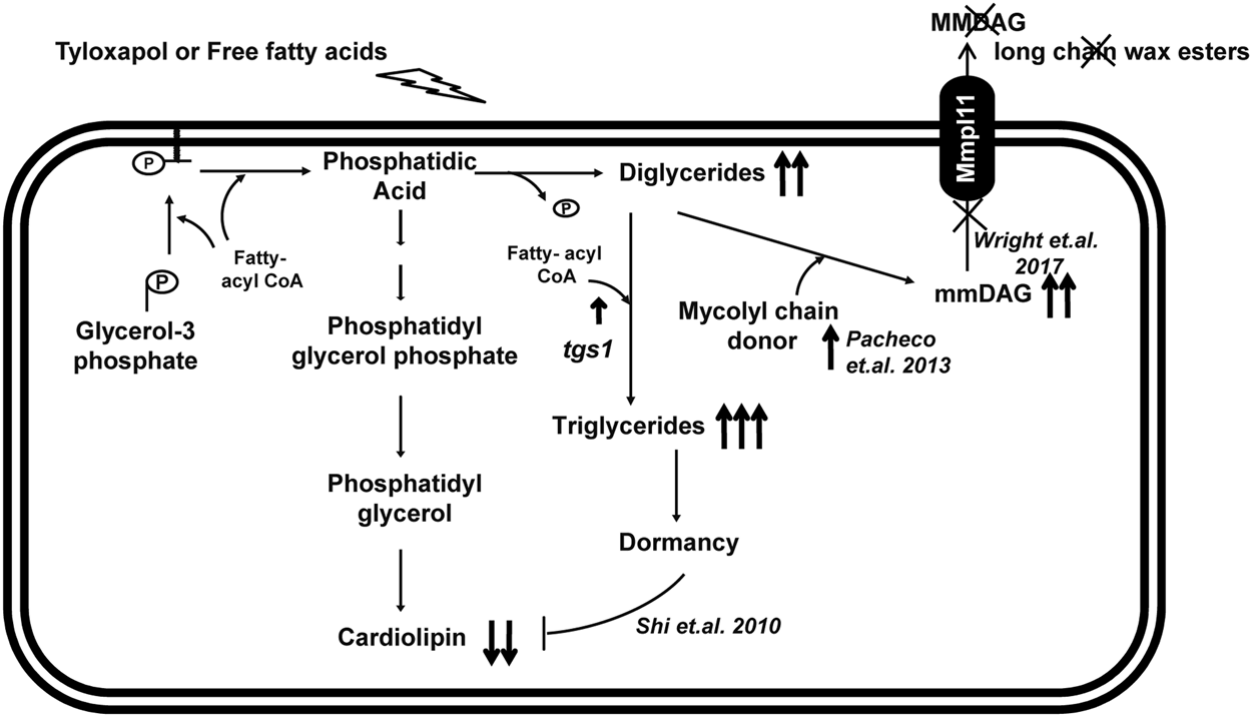
A proposed model for the flux of metabolites controlled by Mtb MmpL11 is depicted.

We here describe a novel function of the Mtb MmpL11 in maintaining cellular homeostasis and integrity in the presence of membrane altering stress (Fig. 6). This is crucial for Mtb survival in the face of extracellular stress like fatty acids, antibiotics and in intracellular lipid rich conditions. We propose an important role for Mtb MmpL11 on Mtb’s capability to grow in conditions of excessive fatty acid presence in the milieu as would be seen *in-vivo* in caseating/ necrotising granulomas.

## Methods

### Bacterial Strains and Growth Conditions

*M. tuberculosis* Erdman (Wt), a kind gift of Dr. Jeffrey Cox, USA, ΔM11 and complemented strains were grown in Middlebrook 7H9 broth (BD Biosciences, USA) and 0.5% glycerol in either the presence (7H9c) or absence (7H9g) of 4% acid-albumin-dextrose-catalase (ADC) supplement as required. Growth media was supplemented with 0.05% Tween 80/Tyloxapol or different concentrations of fatty acids methyl esters when required; and on solid media (Middlebrook 7H10 agar, BD Biosciences, USA) supplemented with 0.5% glycerol and 10% OADC (BD Biosciences, USA). Standard procedures were used for culturing *E.coli* strains in LB broth or agar (BD Biosciences, USA). Media was supplemented with kanamycin (20–50 µg/ml), hygromycin (50–150 µg/ml) or carbenicillin (100 µg/ml) when needed.

### Construction of *M. tuberculosis mmpL11* null mutant

The *mmpL11* null mutant (ΔM11) was created by homologous recombination using specialized transduction and recombineering^55^. The 5′ flanking region and 3′ flanking region of *mmpL11* (Rv0202c) was amplified from Mtb genomic DNA using specific primers (Table 1) and phusion DNA polymerase (Thermo Fisher Scientific Inc., USA) and cloned in pJET1.2 cloning vector (Thermo Fisher Scientific Inc, USA). After confirmation by sequencing, the flanks were cloned serially into pMSG360 to obtain the final plasmid pANK64. An allelic exchange fragment on *AflII* and *DraI* digestion of pANK64 was used as a template for recombineering mediated generation of phages that were used for transduction of Wt Erdman Mtb cultures. Hyg^R^ colonies obtained were screened for deletion of *mmpL11* by Southern hybridization and qPCR as per recommended protocols. For complementation, the complete *mmpL11* was amplified and cloned in the mycobacterial integration vector- pTC01-XL; expression was confirmed by gene specific qPCR as per standard protocols.

**Table 1:** List of primers used in the study.

Name	Sequence	Reference
M11 5′ F	GCACTAGTCTGATCGGCAGCGAGCAC	This study
M11 5′ R	GGAAGCTTTGCGCAGGTTGCGGCTC	This study
M11 3′ F	GGTCTAGAGTGGCCGTGGCGATGTTGG	This study
M11 3′ R	GCGGTACCGGTGATTCTGCGGGTTAGC	This study
P 1	GCATCTACCGGCGGGCGCCCTGC	This study
P 2	GTGTTACGGCCCAGGCCGCTACG	This study
M11 Comp F	GACATATGCCTCGCCTCCTCCAACATC	This study
M11 Comp R	GCCATATGATGCGCTTGAGCC	This study
(*rrs*)16 S RT F	ATGACGGCCTTCGGGTTGTAA	This study
(*rrs*)16 S RT R	CGGCTGCTGGCACGTAGTTG	This study
*rpoB* RT F	CGACGAGTGCAAAGACAAGG	This study
*rpoB* RT R	TCGGGAAGTCACCCATGAAC	This study
M11 RT F	AATGCCGTCGCGCTACTGC	This study
M11 RT R	TTGCCGTTGGCAGTTTGCC	This study
*tgs1* RT F	CGATCCCGACTTCGATCTTG	This study
*tgs1* RT R	AGGCCTTCGATGACCCAGAC	This study
RV2881c RT R	ACCTCATGGGTAGCGACCAAGG	This study
Rv2881c RT F	GCGCGTGGTGCTAAACAGCAG	This study
Rv2746c RT R	TGCCATCCCGTAATTGCGAGCC	This study
Rv2746c RT F	TTGCCGTGTCGGCGCAGCCTG	This study
Rv2612c RT R	AACACCGCGCCGTCACTGATGC	This study
Rv2612c RT F	ACGCCGGACGTCGTCACCATC	This study
RV1822 RT R	AGGCGATCAACGGCCGGGTCC	This study
Rv1822 RT F	ATTCGCCTCGCGCTCATCCCAG	This study
Rv0436c RT F	AGCGCGATGACGGTGCTGTCC	This study
Rv0436c RT R	GACACGTAAAGCACCAGCGCG	This study
*lysX* RT R	CCTGGTGGCCGAGCGAGGTTTG	This study
*lysX* RT F	GGGATGGTCGCGGCGTCCAGTC	This study
*devR* F	CCGATCTGCGCTGTCTGATC	^52^
*devR* R	GTCCAGCGCCCACATCTTT	^52^
*narX* RT F	ATGATGGGCGAACTCTTCTG	This study
*narX* RT R	CAGCCGAATTTGTCATAGCG	This study
*narK2* RT F	GACCTGGGAGATGTCGTTTC	This study
*narK2* RT R	TGATGTAGGTGGGCAGGTAG	This study

### Analysis of gene expression by qPCR

Cells were grown either in 0.1% Tween 80 or Tyloxapol for 24 hr in 7H9 minimal media containing 0.5% glycerol at 37 °C. Total RNA was isolated by bead beating the cultures in Trizol (Invitrogen, Thermo Fisher Scientific Inc., USA). cDNA synthesis was done from 1 µg of RNA by using Verso cDNA synthesis kit (Fermentas, Thermo Fisher Scientific Inc., USA), and used for gene specific qPCR with 5 ng of cDNA in LC480 (Roche diagnostics, USA).

### Stress dependent growth of Mtb

The three Mtb strains were subjected to *in vitro* stress conditions in minimal 7H9 media or containing 0.5% dextrose, 0.5% glycerol, methyl ester of fatty acids, surfactants like Tween 80, Tyloxapol, Triton X-100, IGEPAL CA-630, H_2_O_2_ (in the range of 100 mM to 5 mM) or DETA-NO (from 6.25–50 µM) respectively (Sigma-Aldrich Corp., USA). The strains were grown in 96-well plates at 37 °C and growth was monitored by measuring absorbance at 600 nm. For testing pH dependent growth, strains were grown in Sauton’s media containing 0.05% Tyloxapol instead of Tween 80.

### Transmission Electron Microscopy

Transmission electron microscopy (TEM) analysis of Erdman, ΔM11 and Comp grown in the presence of Tween 80 or Tyloxapol was done by using standard protocols. Briefly, cells fixed in 2.5% gluteraldehyde and 4% paraformaldehyde and dehydrated in graded series of alcohol were embedded in the Epon 812 resin. Ultrathin sections were cut and stained with uranyl acetate and lead citrate. TEM images were captured using Tecnai G2 20 twin (FEI) transmission electron microscope (FEI, Thermo Fisher Scientific Inc., USA).

### Confocal Microscopy and Image analysis

The fluorescent acid-fast staining dye Auramine O (AO) was used in combination with Nile Red as per standard protocols to stain cultures treated for 24 hr with Tween 80 or Tyloxapol^32^. Briefly, each sample was stained with 0.2% fluorescent AO (in 4% phenol solution) for 15 min, washed and treated with decolorizing solution for 5 min and finally stained with 30 µg/ml of NR for 15 min. The samples were then fixed on microscopic slides and imaged on Lieca TCS SP8 Confocal Microscope (Leica Microsystems, USA). For microscopic examination of cardiolipin, cells from 16 hr detergent treated cultures were stained directly in the growth medium with 200 nM NAO (10-N-Nonyl acridine orange) for 30 min at 37 °C in presence of Tween 80 or Tyloxapol, fixed and used for imaging. Extent of staining was quantitated by using VOLOCITY image analysis software (PerkinElmer, Ohio, USA).

### Membrane permeability and polarity assays

The membrane potential of Mtb grown in Tween 80 or Tyloxapol was analyzed using DiOC_2_(3) as per standardized protocols^56^. Briefly, 10^6^ cells were stained with 30 µM dye for 30 min in growth media, the excess dye was removed by washing with PBS, fixed with 2% PFA and analyzed by FACS in a BD LSRII (BD Biosciences, USA). For permeability assays using Hoechst-33342, 1.5 × 10^8^ cells were stained with 2.5 µM dye for 30 min in growth media at 37 °C. The fluorescence at ex355nm/ em460nm was measured in a Tecan Infinite M200 pro spectrophotometer (Tecan Gr. Ltd., Switzerland).

### Analysis of lipids from cells treated with Tween 80 and Tyloxapol

Logarithmic grown cells were washed twice with 1X -PBS and re-suspended in either 10 ml media containing 4% ADC and 0.5% glycerol or minimal media with 0.5% glycerol only and supplemented with either 0.1% Tween 80 or Tyloxapol. After 18 hr of growth at 37 °C, 3 µCi of ^14^C-Oleate sodium salt (American Radiolabeled Chemicals, Inc. USA), were added and the extent of radiolabel incorporation after 6 hr into the different lipid fractions were calculated by using TopCount NXT scintillation counter (PerkinElmer, Ohio, USA). Equal counts of 10,000 cpm (counts per minute) were spotted on TLC silica gel 60 (Merck Millipore, USA) for all three strains and eluted using CHCl_3_:CH_3_OH:H_2_O_2_::65:25:4 for phospholipids and petroleum ether:diethyl ether:acetic acid::70:30:1 for TAG as the solvent. The TLCs were scanned using GE Typhoon FLA 7000 phosphorimager system (GE Healthcare Bio-Sciences, USA) quantitated by densitometry using IQ50. Total polar and apolar lipid fractions were isolated from 16 hr ^14^C-acetate labeled cultures and analyzed by TLC according to previously published protocols^57^. Methyl esters of fatty acids and mycolic acids were isolated from the Mtb cultures labeled with ^14^C-propionate and analyzed by TLC as per established protocols^58^.

### SPE column fractionation and Mass spectrometric analysis of phosphatidyl glycerol or cardiolipin

Phospholipids were fractionated using aminopropyl-silica gel cartridges (SPE columns, Phenomenox, USA) as described previously^59^. Briefly, total lipids from Mtb strains grown in 0.1% Tween 80 or Tyloxapol containing media for 24 hr were extracted using CHCl_3_:CH_3_OH:H_2_O::2:1:0.1. The extracts were dried at 60 °C and re-suspended in 200 μl of cyclohexane:CHCl_3_:CH_3_OH::95:3:2 and allowed to bind to a pre-equilibriated SPE column for 10 min. Subsequently, fraction I containing all neutral lipids was eluted with 10 ml of a solvent mixture containing petroleum ether:diethyl ether:acetic acid::80:20:3. Fraction II (containing PE, majorly) was eluted with 3 ml of CHCl_3_:CH_3_OH::2:1. Fraction III (containing PG and CL) was eluted with 10 ml of CHCl_3_:CH_3_OH:NH_4_OH::4:1:0.1. Fractions IV and V were eluted with CHCl_3_:CH_3_OH::4:1 containing 0.05 M and 0.2 M NH_4_CH_3_CO_2_, respectively. Individual samples were resolved on TLC slica gel 60 in the CHCl_3_:CH_3_OH:H_2_O::65:25:4 solvent system. For ESI- MS analysis the purified Fractions- III and IV from treated cultures were subjected to MS and MS2 analysis in an AB SCIEX Triple TOF 5600 mass spectrometer (AB SCIEX LP, Canada).

### Infection of THP1 macrophages

THP1 cells were grown in HiglutaXL RPMI-1640 (Himedia laboratories, India) with 10% FBS and seeded in 48-well plates at a density of 1.5 × 10^5^ cells per well. Following differentiation with 100 nM PMA for 24 hr (Sigma-Aldrich Corp., USA) and additional 48 hr without PMA, cells were infected with single cells suspensions (SCS) at an MOI of 5 of individual or 1:1 mixed density of Wt and ΔM11 for 6 hr. After washing extracellular bacteria, intracellular bacterial burdens were estimated by serial dilution plating of cell lysates on 7H10 agar plates. Bacterial colony numbers obtained after 3–5 weeks of incubation at 37 °C is represented as cfu/ml. For estimating the relative growth fitness of ΔM11, colonies obtained by plating identical volumes of cell lysates in the presence of 50 µg/ml hygromycin (Hyg^R^-ΔM11) or without antibiotic were enumerated and used to calculate relative fitness *i.e*.- Hyg^R^ cfu/ml / [(total cfu/ml)-(Hyg^R^ cfu/ml)]. For growth in lipid rich macrophages, THP1 cells were pre-treated with 200 µM oleic acid (Sigma-Aldrich Corp., USA) for 16 hr and then infected with the Mtb strains.

For fatty acid uptake studies, cells at a density of 1.5 × 10^5^ cells per well were seeded on confocal coverslips. Following differentiation as described earlier, cells were infected with a single cell suspension (SCS) of Hoechst-33342 stained Wt and ΔM11 at density of MOI of 50 for 3 hr. Extracellular bacteria were removed by washing and the cells were labelled with 1 µm of fluorescent BODIPY™ 558/568 C_12_ fatty acid (Thermo Fisher Scientific Inc., USA) for 1 hr. The samples were then fixed in 4% formaldehyde after removal of excess extracellular dye and imaged and analyzed on Lieca TCS SP8 Confocal Microscope (Leica Microsystems, USA).

### Statistical Analysis

All experiments were performed multiple times. Statistical analysis was done by using Student’s t-test. * represents p-value < 0.05, ** represents the p-value < 0.01 and *** represents the p-value < 0.005.

## Electronic supplementary material

Supplementary figures

## References

[1] Barkan D, Liu Z, Sacchettini JC, Glickman MS (2009). Mycolic acid cyclopropanation is essential for viability, drug resistance, and cell wall integrity of Mycobacterium tuberculosis. Chem Biol.

[2] Rao V, Fujiwara N, Porcelli SA, Glickman MS (2005). Mycobacterium tuberculosis controls host innate immune activation through cyclopropane modification of a glycolipid effector molecule. J Exp Med.

[3] Fu YR, Gao KS, Ji R, Yi ZJ (2015). Differential transcriptional response in macrophages infected with cell wall deficient versus normal Mycobacterium Tuberculosis. Int J Biol Sci.

[4] Erdemli, S. B. *et al*. Targeting the cell wall of Mycobacterium tuberculosis: structure and mechanism of L,D-transpeptidase 2. *Structure* **20**, 2103-2115. 10.1016/j.str.2012.09.016 PMC3573878 23103390

[5] Abrahams KA, Besra GS (2018). Mycobacterial cell wall biosynthesis: a multifaceted antibiotic target. Parasitology.

[6] Andries K (2014). Acquired resistance of Mycobacterium tuberculosis to bedaquiline. PLoS One.

[7] Szumowski JD, Adams KN, Edelstein PH, Ramakrishnan L (2013). Antimicrobial efflux pumps and Mycobacterium tuberculosis drug tolerance: evolutionary considerations. Curr Top Microbiol Immunol.

[8] Garima K (2015). Differential expression of efflux pump genes of Mycobacterium tuberculosis in response to varied subinhibitory concentrations of antituberculosis agents. Tuberculosis (Edinb).

[9] Szekely R, Cole ST (2016). Mechanistic insight into mycobacterial MmpL protein function. Molecular microbiology.

[10] Viljoen A (2017). The diverse family of MmpL transporters in mycobacteria: from regulation to antimicrobial developments. Molecular microbiology.

[11] Converse SE (2003). MmpL8 is required for sulfolipid-1 biosynthesis and Mycobacterium tuberculosis virulence. Proc Natl Acad Sci USA.

[12] Varela C (2012). MmpL genes are associated with mycolic acid metabolism in mycobacteria and corynebacteria. Chem Biol.

[13] Wells, R. M. *et al*. Discovery of a siderophore export system essential for virulence of Mycobacterium tuberculosis. *PLoS pathogens* **9**, (2013). 10.1371/journal.ppat.1003120 PMC3561183 23431276

[14] Jones, C. M. *et al*. Self-poisoning of Mycobacterium tuberculosis by interrupting siderophore recycling. *Proc Natl Acad Sci USA* **111**, (2014). 10.1073/pnas.1311402111 PMC3918798 24497493

[15] Belardinelli JM (2014). Biosynthesis and translocation of unsulfated acyltrehaloses in Mycobacterium tuberculosis. J Biol Chem.

[16] Chalut C (2016). MmpL transporter-mediated export of cell-wall associated lipids and siderophores in mycobacteria. Tuberculosis (Edinb).

[17] Pacheco, S. A., Hsu, F. F., Powers, K. M. & Purdy, G. E. MmpL11 protein transports mycolic acid-containing lipids to the mycobacterial cell wall and contributes to biofilm formation in Mycobacterium smegmatis. *J Biol Chem* **288**, (2013). 10.1074/jbc.M113.473371 PMC3745366 23836904

[18] Wright, C. C. *et al*. The Mycobacterium tuberculosis MmpL11 Cell Wall Lipid Transporter Is Important for Biofilm Formation, Intracellular Growth, and Nonreplicating Persistence. *Infection and immunity* **85**, (2017). 10.1128/IAI.00131-17 PMC5520431 28507063

[19] Degiacomi G (2017). Essentiality of mmpL3 and impact of its silencing on Mycobacterium tuberculosis gene expression. Sci Rep.

[20] Domenech P, Reed MB, Barry CE (2005). Contribution of the Mycobacterium tuberculosis MmpL protein family to virulence and drug resistance. Infect Immun.

[21] Goren MB, Brokl O, Schaefer WB (1974). Lipids of putative relevance to virulence in Mycobacterium tuberculosis: phthiocerol dimycocerosate and the attenuation indicator lipid. Infection and immunity.

[22] Cox JS, Chen B, McNeil M, Jacobs WR (1999). Complex lipid determines tissue-specific replication of Mycobacterium tuberculosis in mice. Nature.

[23] Rousseau C (2004). Production of phthiocerol dimycocerosates protects Mycobacterium tuberculosis from the cidal activity of reactive nitrogen intermediates produced by macrophages and modulates the early immune response to infection. Cellular microbiology.

[24] Astarie-Dequeker C (2009). Phthiocerol dimycocerosates of M. tuberculosis participate in macrophage invasion by inducing changes in the organization of plasma membrane lipids. PLoS pathogens.

[25] McDonnell G, Russell AD (1999). Antiseptics and disinfectants: activity, action, and resistance. Clin Microbiol Rev.

[26] Manganelli R (2004). Sigma factors and global gene regulation in Mycobacterium tuberculosis. Journal of bacteriology.

[27] Stallings CL, Glickman MS (2010). Is Mycobacterium tuberculosis stressed out? A critical assessment of the genetic evidence. Microbes and infection.

[28] Manganelli R, Voskuil MI, Schoolnik GK, Smith I (2001). The Mycobacterium tuberculosis ECF sigma factor sigmaE: role in global gene expression and survival in macrophages. Molecular microbiology.

[29] de Carvalho LP, Darby CM, Rhee KY, Nathan C (2011). Nitazoxanide Disrupts Membrane Potential and Intrabacterial pH Homeostasis of Mycobacterium tuberculosis. ACS Med Chem Lett.

[30] Halder S (2015). Alteration of Zeta potential and membrane permeability in bacteria: a study with cationic agents. Springerplus.

[31] Charbon G (2017). Re-wiring of energy metabolism promotes viability during hyperreplication stress in E. coli. PLoS Genet.

[32] Shi L (2010). Carbon flux rerouting during Mycobacterium tuberculosis growth arrest. Molecular microbiology.

[33] Crellin, P. K., Luo, C.-Y. & Morita, Y. S. In *Lipid* Metabolism (ed Prof. Rodrigo Valenzuela Baez) 119–148 (InTech, 2013).

[34] Daniel J, Maamar H, Deb C, Sirakova TD, Kolattukudy PE (2011). Mycobacterium tuberculosis uses host triacylglycerol to accumulate lipid droplets and acquires a dormancy-like phenotype in lipid-loaded macrophages. PLoS pathogens.

[35] Daniel J, Sirakova T, Kolattukudy P (2014). An acyl-CoA synthetase in Mycobacterium tuberculosis involved in triacylglycerol accumulation during dormancy. PLoS One.

[36] Low KL (2009). Triacylglycerol utilization is required for regrowth of *in vitro* hypoxic nonreplicating Mycobacterium bovis bacillus Calmette-Guerin. J Bacteriol.

[37] Fattorini L, Piccaro G, Mustazzolu A, Giannoni F (2013). Targeting dormant bacilli to fight tuberculosis. Mediterr J Hematol Infect Dis.

[38] Rodriguez JG (2014). Global adaptation to a lipid environment triggers the dormancy-related phenotype of Mycobacterium tuberculosis. MBio.

[39] Berrington WR, Hawn TR (2007). Mycobacterium tuberculosis, macrophages, and the innate immune response: does common variation matter?. Immunol Rev.

[40] Lerner TR, Borel S, Gutierrez MG (2015). The innate immune response in human tuberculosis. Cell Microbiol.

[41] Santucci P (2016). Experimental Models of Foamy Macrophages and Approaches for Dissecting the Mechanisms of Lipid Accumulation and Consumption during Dormancy and Reactivation of Tuberculosis. Front Cell Infect Microbiol.

[42] Seiler P (2003). Cell-wall alterations as an attribute of Mycobacterium tuberculosis in latent infection. J Infect Dis.

[43] Ortalo-Magne A (1996). Identification of the surface-exposed lipids on the cell envelopes of Mycobacterium tuberculosis and other mycobacterial species. J Bacteriol.

[44] Jankute M, Cox JA, Harrison J, Besra GS (2015). Assembly of the Mycobacterial Cell Wall. Annu Rev Microbiol.

[45] Akella A, Deshpande SB (2013). Pulmonary surfactants and their role in pathophysiology of lung disorders. Indian J Exp Biol.

[46] Srivastava S (2015). Unsaturated Lipid Assimilation by Mycobacteria Requires Auxiliary cis-trans Enoyl CoA Isomerase. Chem Biol.

[47] Morrisett JD, Pownall HJ, Gotto AM (1975). Bovine serum albumin. Study of the fatty acid and steroid binding sites using spin-labeled lipids. The Journal of biological chemistry.

[48] Yadav, P. K. & Rajasekharan, R. Cardiolipin deficiency causes triacylglycerol accumulation in Saccharomyces cerevisiae. *Mol Cell Biochem*10.1007/s11010-017-3039-4 (2017). 10.1007/s11010-017-3039-4 28432553

[49] Garton NJ (2008). Cytological and transcript analyses reveal fat and lazy persister-like bacilli in tuberculous sputum. PLoS Med.

[50] Daniel J, Kapoor N, Sirakova T, Sinha R, Kolattukudy P (2016). The perilipin-like PPE15 protein in Mycobacterium tuberculosis is required for triacylglycerol accumulation under dormancy-inducing conditions. Molecular microbiology.

[51] Chauhan S, Tyagi JS (2009). Powerful induction of divergent tgs1-Rv3131 genes in Mycobacterium tuberculosis is mediated by DevR interaction with a high-affinity site and an adjacent cryptic low-affinity site. J Bacteriol.

[52] Taneja NK, Dhingra S, Mittal A, Naresh M, Tyagi JS (2010). Mycobacterium tuberculosis transcriptional adaptation, growth arrest and dormancy phenotype development is triggered by vitamin C. PLoS One.

[53] Voskuil MI, Bartek IL, Visconti K, Schoolnik GK (2011). The response of mycobacterium tuberculosis to reactive oxygen and nitrogen species. Front Microbiol.

[54] Cotes K (2008). Lipolytic enzymes in Mycobacterium tuberculosis. Appl Microbiol Biotechnol.

[55] Barkan D, Rao V, Sukenick GD, Glickman MS (2010). Redundant function of cmaA2 and mmaA2 in Mycobacterium tuberculosis cis cyclopropanation of oxygenated mycolates. J Bacteriol.

[56] Anand A (2015). Polyketide Quinones Are Alternate Intermediate Electron Carriers during Mycobacterial Respiration in Oxygen-Deficient Niches. Molecular cell.

[57] Slayden RA, Barry CE (2001). Analysis of the Lipids of Mycobacterium tuberculosis. Methods Mol Med.

[58] Vilcheze, C. & Jacobs, W. R. Isolation and analysis of Mycobacterium tuberculosis mycolic acids. *Curr Protoc Microbio*l Chapter 10, Unit10A 13, 10.1002/9780471729259.mc10a03s05 (2007). 10.1002/9780471729259.mc10a03s05 18770604

[59] Fauland A (2013). An improved SPE method for fractionation and identification of phospholipids. J Sep Sci.

