# Supplementary material for: Phospholipid homeostasis, membrane tenacity and survival of Mtb in lipid rich conditions is determined by MmpL11 function
Source: Sci Rep. 2018 May 29;8:8317. doi: 10.1038/s41598-018-26710-z (PMC5974182; doi:10.1038/s41598-018-26710-z)
Supplement: Supplementary file 1 — Supplementary figures [file 41598_2018_26710_MOESM1_ESM.pdf]

Phospholipid homeostasis, membrane tenacity and survival of Mtb in lipid rich conditions is determined by MmpL11 function

Ankur Bothra[1], Prabhakar Arumugam[1], Vipul Panchal[1], Dilip Menon[1], Sonali Srivastava[1], Deepthi Shankaran[1], Ananya Nandy[1], Neetika Jaisinghani[1], Archana Singh[1], Rajesh S. Gokhale[1,2,+], Sheetal Gandotra[1,2], Vivek Rao[1,2,*]

[1]CSIR- Institute of genomics and Integrative Biology, New Delhi-110025, India

[2]Academy of Scientific and Innovative Research, CSIR-Central Road Research Institute, New Delhi-110025, India (AcSIR)

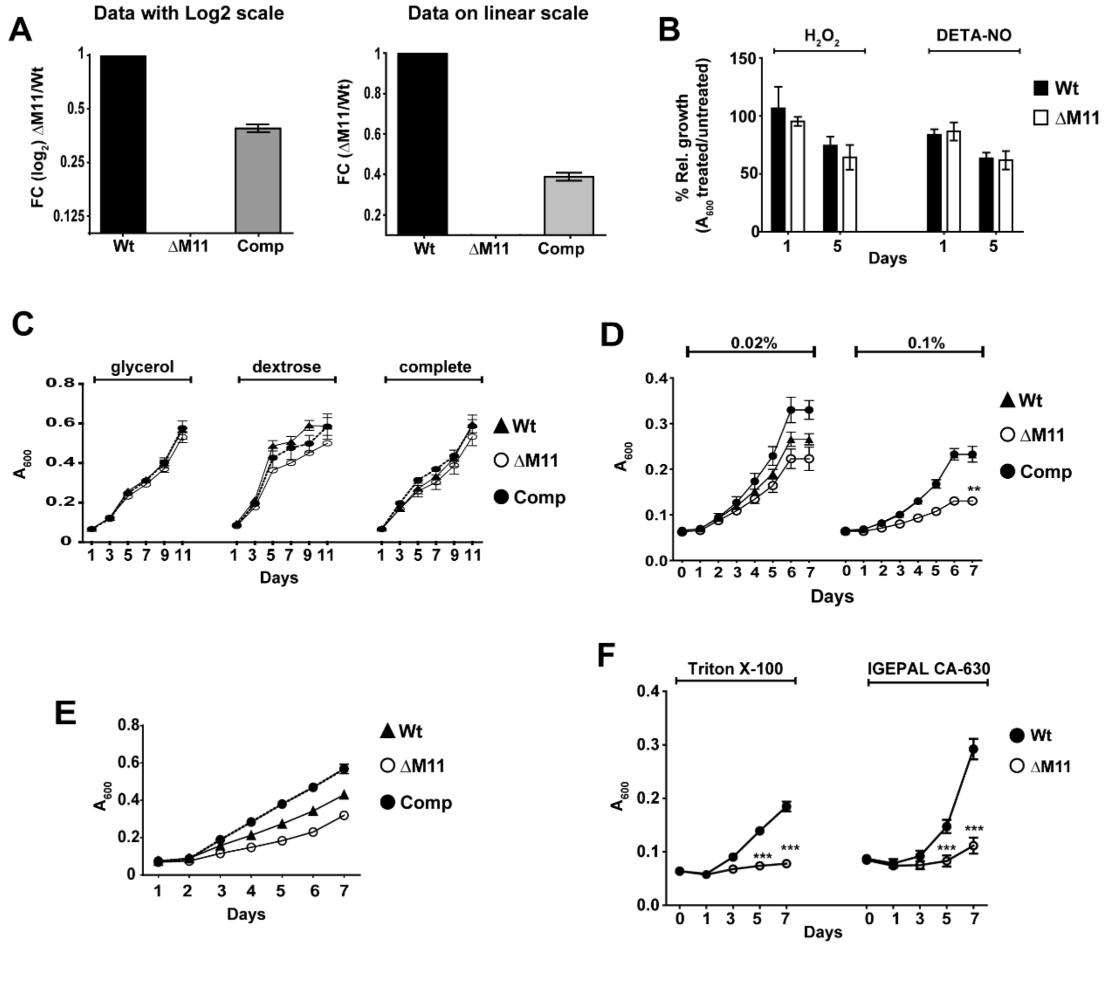

Figure S1

Fig. S1: A) Analysis of *mmpL11* expression in Wt, ΔM11, Comp by qPCR. Gene expression normalised to *rpoB* is plotted as mean ± SD. B-E) Growth of the Mtb strains in 7H9 media- (B) containing 0.5% glycerol or 0.5% dextrose or in complete media containing ADC, (C) after treatment with H_20_2 or DETA-NO for 24 and 120 hrs. (D) containing 0.02% or 0.1% Tyloxapol and (E) containing 0.05% Tyloxapol + ADC. F) Growth of Wt and ΔM11 in 7H9 media containing 0.005% Triton X-100 and 0.05% IGEPAL CA-630 as indicated. Values are plotted as mean of triplicate values ± SD for one representative experiment of n=2 or 3

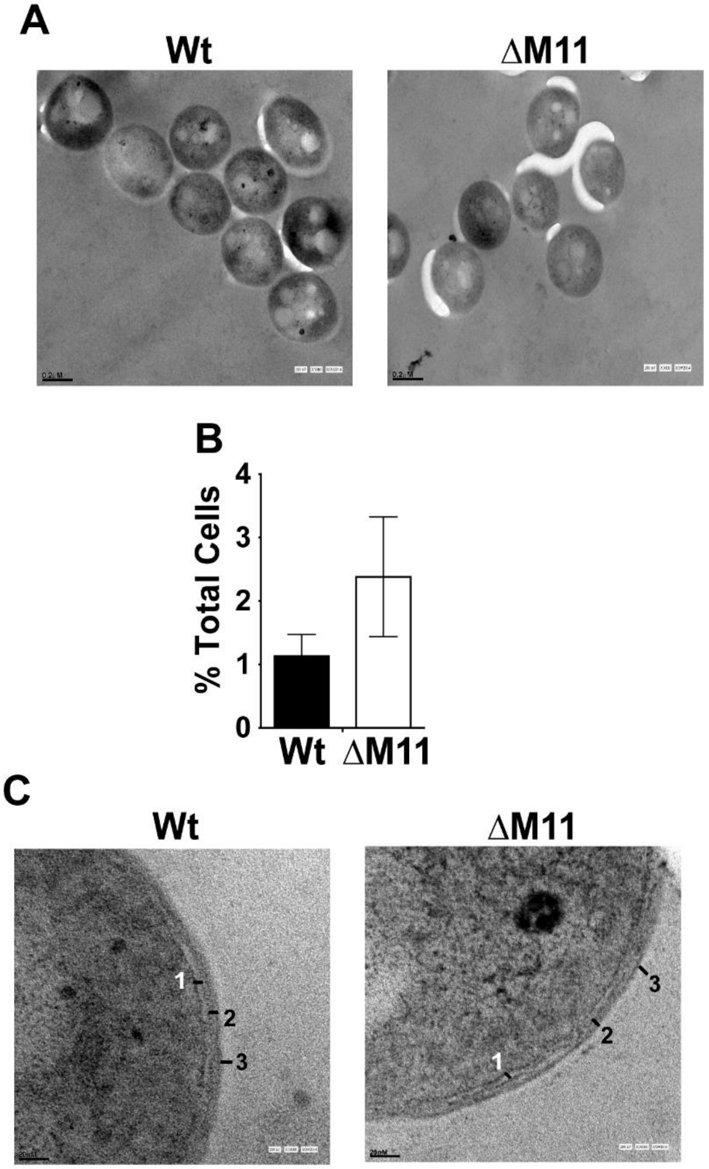

Fig. S2: A, C) Morphology of Wt and ΔM11 strains grown in 0.05% Tween 80 containing media in TEM. Representative images from 3 independent experiments and Scale bars of 0.2 µm and 20 nm are indicated. (B) Quantitation of deformed cells in multiple images of Wt and ΔM11 is graphically represented as mean ± SD.

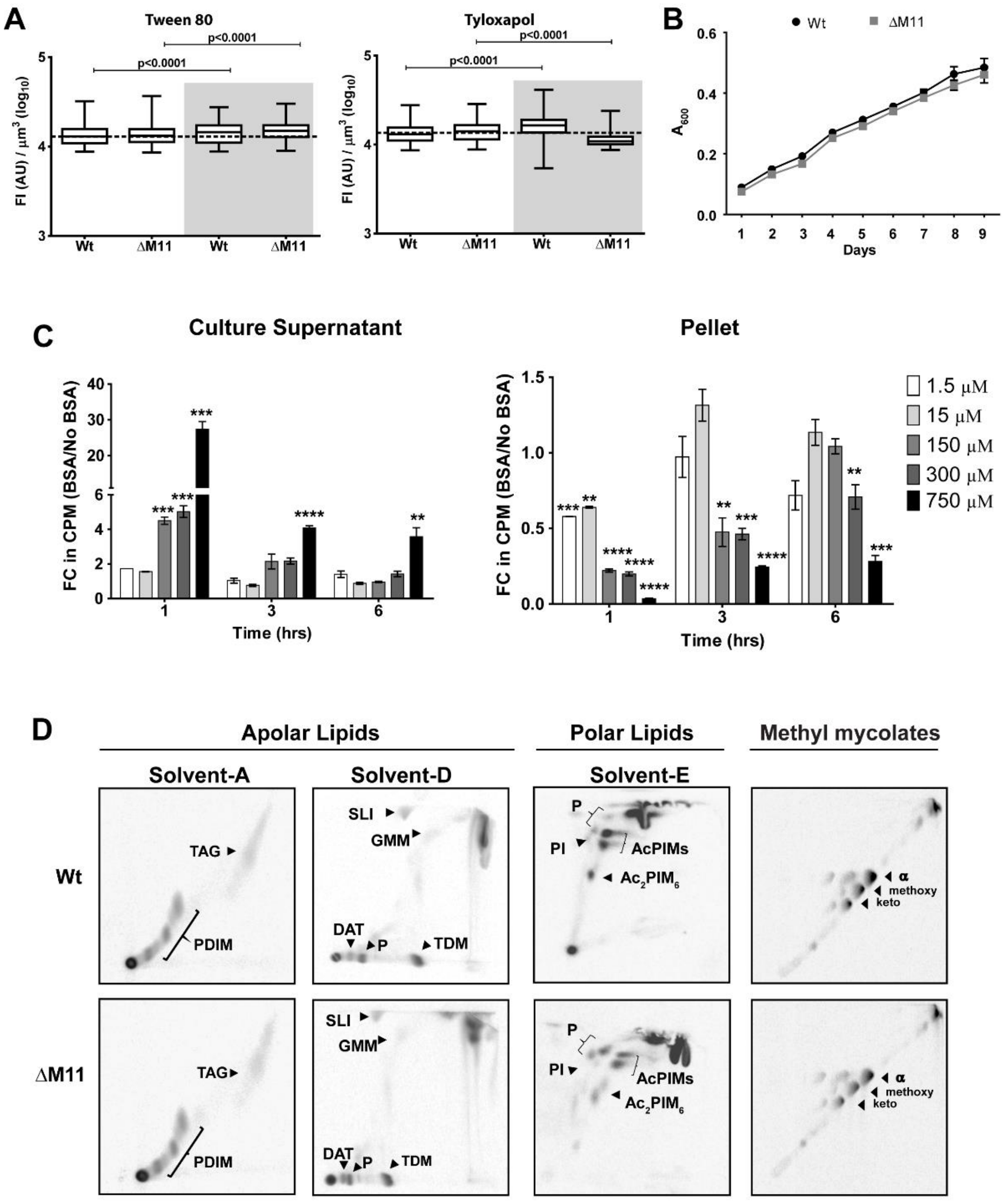

Fig.S3

Fig. S3: A) Quantitation of CL in Wt and ΔM11 logarithmic cultures grown in the presence of 0.05% Tween 80 or 0.05% Tyloxapol. The mean fluorescence intensities at 529nm and 605nm (shaded area) are shown. B) Growth of Wt and ΔM11 Mtb in media supplemented with 5% surfactant for 9 days is represented as mean OD± SD of one representative experiment of n=3. (C) Fatty acid quenching by bovine serum albumin (BSA). Proportion of fatty acid obtained in

culture supernatant containing different concentrations of BSA (in µM) and the bacterial pellet in the respective media are represented as fold change (FC) relative to no BSA containing media at different time intervals of incubation at 37°C. (D) 2D-TLC of ^{14}C-acetate labelled Mtb lipids from logarithmic cultures of Wt and ΔM11. The apolar lipids were resolved with Solvent-A or D while the polar lipids were resolved in solvent-E. Individual lipid species are depicted in the images, PDIM- phthiocerol dimycocerosate, SL1- mature sulfolipid, GMM- glucose monomycolate, DAT- diacyl trehalose, TDM- trehalose dimycolate, P-polar lipids, Ac-PIM- acylated phosphatidyl inositol mannosides. MAMEs and FAMEs were analysed by 2D-TLC with argentation. Individual lipid species are depicted in the images, α- alpha mycolates.

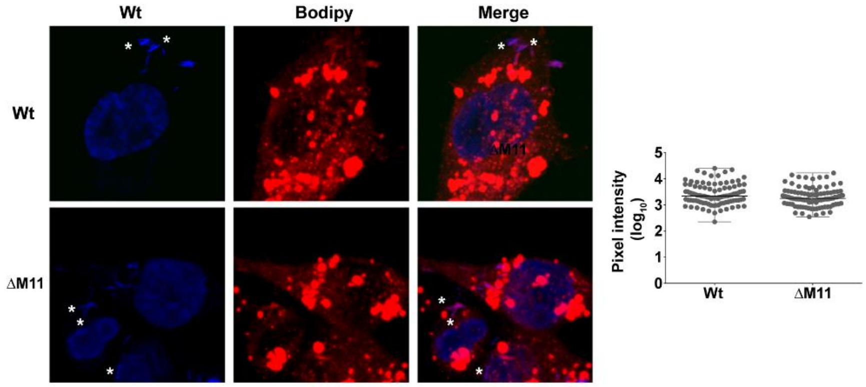

Fig. S4: Estimation of fluorescently labelled BODIPY™ 558/568 C12 fatty acid by Mtb strains in THP1 macrophages (A). The total intensities for 100 bacteria (ROI) was quantitated and is represented graphically (B).
